# Incidence, characteristics, and comorbidities of a complete unselected Danish cohort of patients with thymic epithelial tumors

**DOI:** 10.2340/1651-226X.2025.41295

**Published:** 2025-01-15

**Authors:** Tine Østergaard, Caroline V. A. Bjerke, Eric Santoni-Rugiu, Thomas H. L. Jensen, Katharina A. Perell, René H. Petersen, Peter M. Petersen

**Affiliations:** a Department of Oncology, Copenhagen University Hospital, Copenhagen, Denmark; b Department of Pathology, Copenhagen University Hospital, Copenhagen, Denmark; c Department of Cardiothoracic Surgery, Copenhagen University Hospital, Copenhagen, Denmark; d Department of Clinical Medicine, University of Copenhagen, Copenhagen, Denmark

**Keywords:** Thymoma, thymic carcinoma, epidemiology, autoimmune disease, second cancer

## Abstract

**Background and purpose:**

We report the incidence, characteristics, and comorbidities of the complete unselected Danish cohort of patients with thymic epitheliums (TETs), which may serve as evidence for guiding treatment, surveillance, and counselling of TET patients.

**Patients and methods:**

All patients diagnosed with TETs from January 1st, 2015, to December 31st, 2020, were identified using the Danish Pathology Data Registry. Data on patient characteristics, comorbidities, and tumor histology were collected from electronic medical records available for all patients.

**Results:**

The cohort consisted of 283 patients with a mean age of 64 years (SD: 12). The crude rate was 8.2/1,000,000 TETs annually, thus higher than the age-standardized rates of 4.8/1,000,000 in the WHO World Standard Population and 6.1/1,000,000 in the European Standard Population. Thymomas were diagnosed in 240 patients (85%) (9% type A, 31% AB, 18% B1, 26% B2, 6% B3, 5% micronodular, 0.4% metaplastic, and 5% of unspecified subtype), thymic carcinomas in 39 patients (14%), and thymic neuroendocrine tumors in 4 patients (1.4%). Tumors in Tumour, Node, Metastasis (TNM) stage I were diagnosed in 181 patients (64%) and were mostly thymomas (72%). Prior to TET diagnosis, 91 (32%) patients presented with autoimmune disorders (19% myasthenia gravis) and 82 patients (29%) had at least one cancer diagnosis.

**Interpretation:**

We found a higher incidence of TETs in Denmark than in previous European population-based studies, while reporting a similar distribution of histological types and tumor stages. Furthermore, we found an increased prevalence of autoimmune disorders and cancers in the cohort before TET diagnosis compared to the general population.

## Introduction

Tumors of the thymic epithelium (TETs) are the most common malignant neoplasms of the anterior mediastinum and consist of thymomas, thymic carcinomas (TC), and thymic neuroendocrine tumors (NETs) [1]. Thymomas have the highest incidence of all TETs (75–85%) and are characterized by a more indolent and locally invasive growth pattern [1]. TCs are rarer (14–22% of all TETs) and more aggressive with malignant features comparable to high-grade carcinomas derived from other organs [2]. Thymic NETs are even less frequent, accounting for only 2–5% of all thymic tumors, and are classified with the same nomenclature and criteria as for pulmonary NETs [1]. Clinicians are challenged by the different histological types of TETs with respect to the treatment, surveillance, and counselling of patients. Still, the choice of treatment is often based on low evidence due to the rarity of the diseases and scarcity of easily comparable studies investigating these patients.

Diagnosis of TET is associated with an increased risk of developing autoimmune disorders and secondary cancers [3, 4], which is suspected to involve an immune deficiency caused by dysfunction of the TET [5]. This association is strongest for patients with thymoma, one third of whom are affected by the autoimmune disease myasthenia gravis [3]. Still, no recent studies have presented the incidence of the TET-associated comorbidities in a complete and unselected cohort, thus offering no comprehensive evidence-backed guidance for clinicians in the surveillance and counselling of TET patients.

To address these issues, our study reports the epidemiology of TETs in the complete unselected national Danish cohort, thus providing an unbiased description of the patient population, disease characteristics, and associated comorbidities. The aim of this report is to provide evidence to guide clinicians when planning treatment, surveillance, and counselling of patients diagnosed with TETs and to eventually improve both prognosis and life quality for these patients.

## Patients and methods

This study analyzes data from an entire cohort of 283 Danish TET patients. The inclusion criteria were an established diagnosis of either thymoma, TC, or NET in the Danish Pathology Data Bank (DPDB) between 1st of January 2015 and 31st of December 2020.

The DPDB is an underlying registration database for the Danish National Pathology Registry (DNPR) containing all records of Danish pathology specimens analyzed since 1997, thereby enabling identification of the complete cohort of Danish TET patients with pathologic date of diagnosis between 2015 and 2020 [6]. Given that treatment of TET in Denmark is centralized at our institution, all the final histopathological diagnoses of TET in the study period were made as consensus diagnoses by at least two thorax pathologists from our institution to minimize interobserver variation regardless of whether these diagnoses were made on specimens from our hospital or revision of specimens from other Danish hospitals.

Information concerning the disease, patient characteristics, and cancer diagnoses as well as all entities of autoimmune disorders established prior to TET diagnosis were collected for all patients. Data was collected from electronic medical records available for all patients in SP EPIC, thus providing healthcare information of all hospitalizations for the complete cohort.

All tumors were staged according to the 8^th^ edition of the Union for International Cancer Control (UICC) Tumour, Node, Metastasis (TNM) staging system [7]. Resected tumors were staged pathologically and according to the radicality of the resection, while non-resected tumors were staged clinically based on imaging.

The TETs were histologically classified according to the 5th edition of WHO’s Classification of Thoracic Tumors [1]. For a better overview, mixed TETs with both thymoma and TC histology were registered and biologically considered (therefore, hereby presented) as TCs. For mixed thymomas, we present histology grouped according to the combined components and the representation of each dominant or minor component with 10% intervals in the tumor tissue [1]. For more details on mixed TETs, see Supplementary Table 1.

The age- and sex-standardized incidence rates were calculated using R version 4.3.0 according to IARC’s Cancer Epidemiological Principles and Methods [8]. Age-standardized rates (ASRs) were made in the WHO World Standard Population and European Standard Population [9]. The observed incidence rates were calculated using data on the annual population at risk from the Danish Statistical Register from the 1st of July from 2015 to 2020 [10]. Other statistical analyses were made in IBM SPSS Statistics 25.

## Results

The cohort had a mean age of 64 years (SD: 12) at the time of TET diagnosis (Table 1). More females were observed in the group of patients diagnosed with thymomas (129/240), while more males were in the group of patients diagnosed with TC (25/39) and NETs (4/4) (Supplementary Table 2).

**Table 1:** Characteristics of the 283 Danish patients diagnosed with TETs between 1st of January 2015 and 31st of December 2020.

Patient characteristics	Patients			
	*n*	%	Mean	s.d.
Age, Mean			64	12
Female	143	50.5		
Male	140	49.5		
**Neuroendocrine tumor**	**4**	**1.4**		
Atypical carcinoid	3	1		
Large cell neuroendocrine	1	0.4		
**Thymic carcinoma**	**39**	**14**		
Squamous TC	34	12		
Micronodular TC	2	0.7		
Other TC	3	1.1		
**Thymoma**	**240**	**85**		
A	22	8		
AB	76	27		
B1	44	16		
B2	62	22		
B3	14	5		
Micronodular Thymoma	12	4		
Metaplastic Thymoma	1	0.4		
NOS subtype*	9	3		
**TNM stages**				
I	181	64		
II	31	11		
IIIa	22	8		
IIIb	5	2		
Iva	24	9		
IVb	20	7		
**Comorbidities**				
Autoimmune disorders	91	32		
Previous cancers	82	29		

For data on TETs of mixed subtype, see Supplementary Table 1.

* Not otherwise specified (NOS) subtype due to insufficient tumor tissue. TET: thymic epithelium.

Asymptomatic tumors were diagnosed in 81 patients (29%) and were primarily discovered as incidental findings on computerized tomography (CT) scans performed for other purposes.

**Figure 1:**
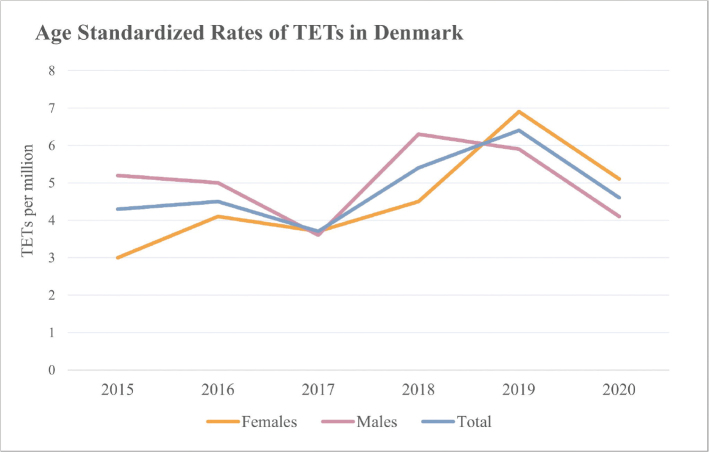
Age standardized rates (ASRs) of TETs in Denmark in the WHO World Standard Population from January 1st, 2015, to December 31st, 2020. For annual incidences, crude-rates, and all incidence-rates of thymomas, TCs and NETs in Denmark, see Supplementary Table 2. TET: thymic epithelium; TC: thymic carcinomas; NET: neuroendocrine tumor.

An average of 47 patients/year were diagnosed with TETs during the time period between 1st of January 2015 and 31st of December 2020, resulting in a crude rate of 8.2/1,000,000 in the Danish population (Supplementary Table 2). The ASR of TETs in Denmark was 4.8/1,000,000 in the WHO World Standard Population (Figure 1) and 6.1/1,000,000 in the European Standard Population during the same time period.

Thymomas were the most common histological category of TETs with an average annual incidence of 40 per year and an ASR of 4/1,000,000 in the WHO World Standard Population. TCs were in average diagnosed in six patients annually resulting in an ASR of 0.7/1,000,000. Only four patients were diagnosed with NETs (Supplementary Table 2).

**Figure 2:**
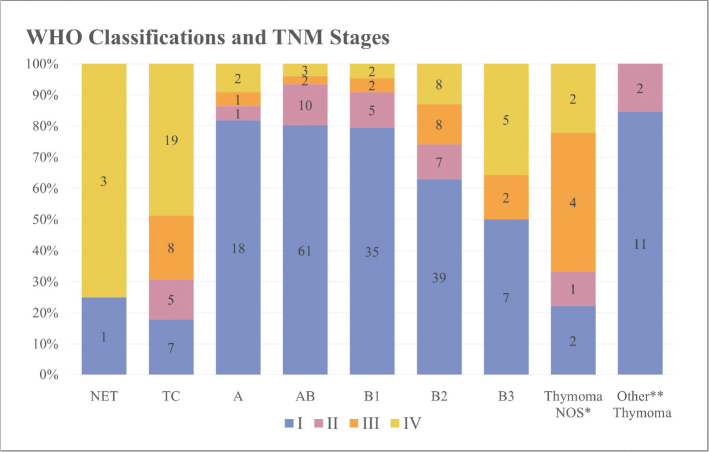
Association between tumor histology and TNM stage. See Supplementary Table 2 for more details on TNM staging of the tumors diagnosed in the cohort. *Not otherwise specified thymomas. The category contains nine thymomas lacking subclassification. Tumor tissue from biopsies proved insufficient for subtype specification in eight of these cases. The last patient was diagnosed with the extremely rare ‘sclerosing thymoma’ (a type no longer present in the current WHO classification of thymomas), which is characterized by extensive sclerosis and inconspicuous areas of preserved thymoma tissue that was unclassifiable. **The category ‘Other Thymomas’ includes 12 micronodular thymomas and a single metaplastic thymoma.

TNM stage I was the most frequent stage of the tumor diagnosed in the cohort (64%), accounting for 72% of the thymomas and 18% of the TCs (Figure 2 and Supplementary Table 3).

**Figure 3:**
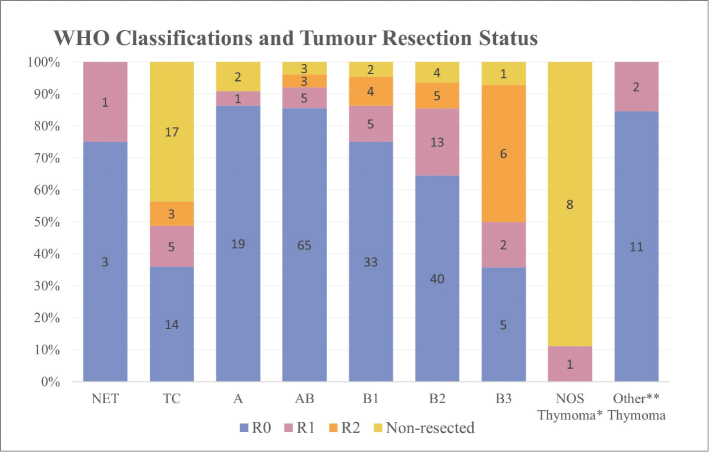
Association between tumor resection status and histologic types. See Supplementary Table 4 for more details on resection status of tumors diagnosed in the cohort. *The category contains nine thymomas lacking subclassification. Tumor tissue from biopsies proved insufficient for subtype specification in eight of these cases. The last patient was diagnosed with the extremely rare ‘sclerosing thymoma’ (a type no longer present in the current WHO classification of thymomas), which is characterized by extensive sclerosis and inconspicuous areas of preserved thymoma tissue that was unclassifiable. **The category ‘Other Thymomas’ includes the 12 micronodular thymomas and a single metaplastic thymoma.

A similar difference was also seen in the surgical resection status as 92% of the patients with thymoma had completely resected tumors (R0) compared to 66% of those with TCs (Figure 3 and Supplementary Table 4).

### Comorbidities

Autoimmune diagnoses were present in 91 (32%) patients prior to TET diagnosis (Table 1). Myasthenia gravis was by far the most frequent autoimmune disorder and was observed in 53 patients (19%). Sporadic cases of other autoimmune disorders of the nervous, endocrine, immune, hematologic, integumentary, or gastrointestinal system were observed in 45 patients (16%) (Table 2 and Supplementary Table 5). Seven patients (2%) had two or more autoimmune disorders at the time of TET diagnosis. Autoimmune diseases were more common in patients with thymomas (36%) than in patients with TCs (15%) or NETs (0%) (Table 2).

**Table 2:** The prevalence of disorders with a suspected autoimmune pathogenesis in patients diagnosed with TETs of various histologic types.

TET histology	Patients with autoimmune disorders	
	*n*	%
Neuroendocrine T	0	0
Thymic Carcinoma	6	15
Thymoma	85	36
A	8	36
AB	21	28
B1	17	39
B2	26	42
B3	9	64
Micronodular Thymoma	2	17
Metaplastic Thymoma	0	0
NOS Subtype	2	22

The prevalence is presented as the proportion of patients with the specific TET type diagnosed with autoimmune diseases. TET: thymic epithelium.

Eighty-two patients (29%) had at least one diagnosis of a non-thymic primary cancer established before the date of TET diagnosis (Table 1), while 10 patients (4%) had two or more cancer diagnoses. Colon and breast cancer were the most frequent extrathymical cancer diagnoses in the cohort and were registered in 21 (7%) and 19 patients (7%), respectively (Table 3).

**Table 3:** Frequency of previous non-thymic primary cancers in the cohort.

Previous cancer diagnoses	Cases	
	*n*	%
Colon cancer	21	7
Breast cancer	19	7
Prostate cancer	9	3
Endometrial cancer	7	2
Lymphoma	6	2
Cervix cancer	6	2
Hematologic cancers	4	1
Melanoma	4	1
Lung cancer	3	1
Urothelial cancer	3	1
Thyroid cancer	2	0.7
Head and neck cancer	2	0.7
Parathyroid cancer*	2	0.7
Renocarcinoma	1	0.4
Germ cell cancer	1	0.4
Pheocromocytoma	1	0.4
Gastric cancer	1	0.4
Malignant nerve sheath tumor	1	0.4

All cancer diagnoses established prior to thymic epithelium (TET) discovery are included except for non-melanoma skin cancers. Twelve patients had two or more established cancer diagnoses before the date of TET diagnosis..

* Two patients were diagnosed with malignant tumors in the parathyroid glands as a manifestation of their Multiple Endocrine Neoplasia (MEN) Syndrome.

## Discussion

This is the first study to report the epidemiology of patients with all types of thymic epithelial tumors in a well-defined and unselected Scandinavian cohort. Data from complete and well-defined cohorts are important to get the full picture of disease characteristics and allow comparison of data from different cohorts as adjustments for patient selection, as incompleteness of cohorts always implies a risk of bias.

Our cohort is complete and with minimal selection bias, because all 283 patients were included through the DPDB with a 100% coverage of malignant diagnoses in Denmark. Since treatment of TETs in Denmark is centralized at our institution, all histopathological diagnoses of TETs during the study period were made as consensus diagnoses on specimens from our hospital and other Danish hospitals by at least two thorax pathologists from our institution. Thereby, diagnostic interobserver variation was minimized. The clinical data is retrieved from electronic medical records available for all patients, thus enabling us to present the true unbiased epidemiology of TETs in a complete Scandinavian cohort.

To our knowledge, the only published study of thymic malignancies in a Scandinavian population was performed by Gadalla et al. and was solely investigating Swedish patients diagnosed with thymomas [11]. From 1958 to 2004, Gardella et al. examined data from 668 Swedish patients with thymomas and reported an increasing trend in the incidence of thymomas from 151 cases (1958–1972) to 315 cases (1988–2004). Still, the average annual incidence of Swedish patients diagnosed with thymomas from 1988 to 2004 (26 patients per year) is notably lower than the incidence of thymomas reported in our study (40 patients/year) (Supplementary Table 2), thereby suggesting a continuous increase in the incidence of thymomas in the Scandinavian population.

The findings of other studies imply a similar increasing trend in the incidence of all TETs. From 1994 to 2003, de Jong et al. reported an ASR of TETs in the Dutch population of 3.2/1,000,000 (in the European standard population), which is notably lower compared to our findings (6.1/1,000,000) [12], thus supporting the hypothesis of a general trend in the European population.

Recent changes in the healthcare systems might explain part of the discrepancy seen in the age-standardized incidence rates of the two studies, as the use of diagnostic imaging has been drastically increasing in the last 20 years [13]. The Danes are among the most CT-scanned populations worldwide [13]. Therefore, the frequent use of CT imaging is expected to contribute to the high ASRs reported in this study and might also explain the notable proportion of asymptomatic tumors (29%) diagnosed in the Danish cohort.

The authors of a more recent study investigating TETs in the South Korean population also believe that the frequent use of CT scans may be causing the increasing trend in the TET incidence [14]. Indeed, in their study Shin et al. reported a steady 6.1% annual increase in the incidence of TETs during the time period from 1999 to 2017, resulting in an increment from 580 patients diagnosed with TETs at the beginning to 2,519 patients diagnosed at the end of that period [14]. This trend is consistent with the difference in incidence of TETs in the Dutch and our Danish study.

Still, the ASR of TETs reported by Shin et al. in South Korea from 2013 to 2017 (7.0/1,000,000 in the WHO World Standard Population) is notably higher than the ASR in Denmark from 2015 to 2020 (4.8/1,000,000) [14]. Hsu et al. presented a possible explanation for this difference in the study of the demographics of the American TET patients, in which they reported the highest incidence of TETs in Asians/Pacific Islanders and the lowest in Caucasians [15], thus suggesting a higher incidence of TETs in Asian populations.

### Histology

The distribution of histological types of TETs in the Danish cohort with 85% thymomas, 14% TCs, and 1.4% NETs resembles that of the Dutch cohort [12]. However, a discrepancy is seen in the distribution of TET types in South Korea with TCs accounting for 38% of the TETs diagnosed in the cohort [14]. The ASR of TCs was correspondingly increased in the South Korea study (2.6/1,000,000 from 2013 to 2017) compared to the Danish ASR of TCs (0.7/1,000,000) [14]. These findings were also in line with the reporting of Hsu et al., finding the incidence of TCs to be significantly increased among American patients with Asian/Pacific Islander heritage [15]. Still, the cause of increased incidence of TCs in the two populations is yet to be understood. In the study, Shin et al. suggest the particularly high exposure to chest radiation for diagnostic or therapeutic purposes to possibly contribute to the increased incidence of TCs in the South Korean population [14]. Although this might explain part of the trend seen in the South Korean population, it is unlikely to explain the increased incidence of TC reported in American patients with Asian/Pacific Islander heritage [15], indicating that ethnical/genetic factors may also play a role.

The distribution of thymoma types in the Danish cohort is concordant with the findings of other studies of European cohorts with thymomas of type AB and B2 as the most frequent [12, 16].

### Staging

A discrepancy was observed in the proportion of patients with localized TETs in Denmark and South Korea. Although reporting a trend towards diagnosis of more TETs in early stage, only 39.2% of the South Korean patients diagnosed in 2017 had localized disease [14], in contrast to 64% of TETs diagnosed in stage I, in our cohort. When compared to the distribution of TNM stages in the Danish cohort, Shin et al. found slightly more patients with regional disease (33.4%), a similar proportion with distant disease (17.9%), and notably more patients with TETs of unknown stage (9%) [14].

### Comorbidities

Autoimmune diseases were reported in cases with a well-established diagnosis. Despite this, even diagnoses of autoimmune diseases collected from electronic patient charts contains some uncertainty; therefore, we suspect underreporting of these disorders in this study.

Still, we find a notably higher prevalence of autoimmune diseases in the cohort prior to TET diagnosis (32%) compared to the general Danish population, assuming a prevalence of autoimmune diseases of 5.29% as reported by Eaton et. al [17].

A few other studies have investigated the chronological development of autoimmune disorders in patients with thymomas with total prevalence of autoimmune diseases in the cohort ranging from 32.7 to 55% [11, 18]. The study performed by Bernard et al. investigated 85 patients diagnosed with thymomas and reported the prevalence of autoimmune disorders to be 38% prior to thymoma diagnosis and 55% after a mean follow-up time of 60 months [18].

Assuming a similar chronology, we expect to see an increase in the prevalence of autoimmune diagnoses in the Danish cohort a few years after TET diagnosis.

We found an increased cumulated risk of non-thymic cancer in TET patients (29%) compared to the cumulative risk of cancer in the Danish 64-year-old individuals between 2015 and 2020 (18%) [19].

Our findings are in concordance with the findings of an American study reporting a significantly increased risk of a second primary cancer in thymoma patients in the SEER database compared to patients with other cancers and of the Swedish study finding the risk of developing secondary primary cancers to be double in patients with thymomas [4, 11]. In concordance with our findings, both studies reported an increased occurrence of breast cancer, colon cancer, prostate cancer, and lymphomas in patients diagnosed with TETs [4, 11]. Still, the incidence order of the specific cancer types varies among the different studies, possibly reflecting differences in genetic and demographic factors as well as lifestyle affecting the risk of developing different extra-thymic cancers in diverse populations [4, 11].

## Conclusion

In conclusion, we succeeded in establishing a complete and unselected cohort of Danish patients diagnosed with TETs, hereby enabling our reporting of the true incidence of TETs in the Danish population. We report a higher incidence of TETs in Denmark compared to the findings of previous European population-based studies, while finding a similar distribution of both histologic types and tumors stages. Lastly, we found the prevalence of autoimmune disorders and cancer diagnoses to be increased in the cohort, even before TET diagnosis, thus providing important information concerning the chronology in the development of the TET-associated comorbidities.

## Supplementary Material

Incidence, characteristics, and comorbidities of a complete unselected Danish cohort of patients with thymic epithelial tumors

## Data Availability

The data presented in this study is not publicly available. The data have been accessed through a study permit from the Capital Region of Denmark allowing the collection (R-20072336 + 21010159) and a permit from the Data Safety Authorities of the Capital Region of Denmark to store these retrospective data concerning the Danish TET patients (P-2020-1132).
