# Supplementary material for: Incidence, characteristics, and comorbidities of a complete unselected Danish cohort of patients with thymic epithelial tumors
Source: Acta Oncol. 2025 Jan 15;64:41295. doi: 10.2340/1651-226X.2025.41295 (PMC11758677; doi:10.2340/1651-226X.2025.41295)
Supplement: Incidence, characteristics, and comorbidities of a complete unselected Danish cohort of patients with thymic epithelial tumors [file AO-64-41295-s1.pdf]

Supplementary material has been published as submitted. It has not been copyedited, or typeset by Acta Oncologica

TETs of Combined Histologic Subtype	
**Mixed TC/Thymoma**	**Patients**
50% B3, 50% TC	1
**Mixed Thymomas**	
90% B1, 10% B2	2
85% B1, 15% B2	1
80% B1, 20% B2	1
70% B1, 30% B2	1
60% B1, 40% B2	1
50% B1, 30% B2, 20% B3	1
90% B2, 10% B1	1
90% B2, 10% B3	1
80% B2, 20% B3	2
80% B2, 20%B3	1
70% B2, 30% B1	1
70% B2, 30% B3	4
70% B2, 20% B1, 10% B3	1
60% B2, 40% B3	2
50% B1, 50% B2	3
100% B3, 100% B1*	
85% B3, 15% B2	1
80% B3, 20% B2	1
70% B3, 30% B2	2
60% B3, 40% B2	2
90% Micronodular, 10% A	1
50% A, 50% Micronodular	1

Supplementary Table 1 *–TETs of mixed histology with their predominant and minor components. A total of 33 patients were diagnosed with TETs of mixed histology.*
For a better overview, thymomas of mixed subtypes were grouped according to the combined components and the representation of each dominant or minor component with 10% intervals in the tumour tissue (1) and are presented as the predominant subtype in our analyses. The TNM stages of the mixed thymomas grouped according to the predominant subtype do not differ significantly from the stages of thymomas with a single histological subtype.
** Two separate fully encapsulated tumours diagnosed in the same patient.*

	Age and Sex Standardized Incidence Rates									
	Female			Male			Total Cohort			
Year of Diagnosis	Annual Incidence	ASR World Standard Population	ASR European Standard Population	Annual Incidence	ASR World Standard Population	ASR European Standard Population	Annual Incidence	Crude Rate	ASR World Standard Population	ASR European Standard Population
**All TETs**										
2015	17	3.4	4.4	24	5.2	6.8	41	7.2	4.3	5.6
2016	21	4.1	5.3	24	5	6.4	45	7.9	4.5	5.9
2017	17	3.7	4.6	15	3.6	4.2	32	5.6	3.7	4.4
2018	23	4.5	5.8	30	6.3	8.2	53	9.2	5.4	7
2019	37	6.9	8.8	27	5.9	7.1	64	11	6.4	8
2020	28	5.1	6.7	20	4.1	5.2	48	8.2	4.6	5.9
**Average**	**24**	**4.6**	**5.9**	**23**	**5**	**6.3**	**47**	**8.2**	**4.8**	**6.1**
**Thymomas**										
2015	16	3.3	4.2	18	3.5	4.7	34	6	3.4	4.5
2016	20	4	5.1	18	3.9	4.9	38	6.6	3.9	5
2017	12	2.8	3.4	10	2.2	2.6	22	3.8	2.5	3
2018	21	3.9	5.1	26	5.4	7	47	8.1	4.6	6
2019	32	6.3	7.9	22	4.7	5.7	54	9.3	5.5	6.8
2020	28	5.1	6.7	15	3.1	3.9	43	7.4	4.1	5.3
**Average**	**22**	**4.2**	**5.4**	**18**	**3.8**	**4.8**	**40**	**6.9**	**4**	**5.1**
**Thymic Carcinomas (TCs)**										
2015	1	0.1	0.2	1	0.1	1.8	6	1.1	0.8	1
2016	1	0.1	0.2	1	0.1	1.1	6	1	0.5	0.7
2017	5	0.9	1.2	5	0.9	1.2	9	1.6	1	1.2
2018	2	0.6	0.7	2	0.6	1	5	0.9	0.7	0.8
2019	5	0.7	0.9	5	0.7	1.1	9	1.5	0.7	1
2020	0	0	0	0	0	1	4	0.7	0.4	0.5
**Average**	**2**	**0.4**	**0.5**	**2**	**0.4**	**1.2**	**6**	**1.1**	**0.7**	**0.9**
**Neuroendocrine Tumours (NETs)**										
2015	0	0	0	1	0.2	0.3	1	0.2	0.1	0.2
2016	0	0	0	1	0.2	0.3	1	0.2	0.1	0.1
2017	0	0	0	0	0	0	0	0	0	0
2018	0	0	0	1	0.2	0.2	1	0.2	0.1	0.1
2019	0	0	0	0	0	0	0	0	0	0
2020	0	0	0	1	0.3	0.3	1	0.2	0.1	0.2
**Average**	**0**	**0**	**0**	**1**	**0.1**	**0.2**	**1**	**0.1**	**0.1**	**0.1**

Supplementary Table 2 *- Annual incidence, crude rates, and age standardized rates (ASRs) of all TETs, thymomas, TCs, and NETs in Denmark from 2015 to 2020. Crude rates and ASRs are presented as tumours per million at risk and the ASRs are calculated in both the WHO world standard population and in the European standard population.*

WHO Classifications and TNM Stages

Classification	I		II		IIIa		IIIb		IVA		IVB		Total	
NET	1	(25%)	0	(0%)	0	(0%)	0	(0%)	3	(75%)	0	(0%)	4	(100%)
TC	7	(18%)	5	(13%)	6	(15%)	2	(5%)	5	(13%)	14	(36%)	39	(100%)
Thymoma	173	(72%)	26	(11%)	16	(7%)	3	(1%)	16	(7%)	6	(3%)	240	(100%)
A	18	(82%)	1	(5%)	0	(0%)	1	(5%)	0	(0%)	2	(9%)	22	(100%)
AB	61	(80%)	10	(13%)	2	(3%)	0	(0%)	1	(1%)	2	(3%)	76	(100%)
B1	35	(80%)	5	(11%)	1	(2%)	1	(2%)	2	(5%)	0	(0%)	44	(100%)
B2	39	(63%)	7	(11%)	8	(13%)	0	(0%)	7	(11%)	1	(2%)	62	(100%)
B3	7	(50%)	0	(0%)	2	(14%)	0	(0%)	4	(29%)	1	(7%)	14	(100%)
Metaplastic Thymoma	1	(100%)	0	(0%)	0	(0%)	0	(0%)	0	(0%)	0	(0%)	1	(100%)
Micronodular Thymoma	10	(83%)	2	(17%)	0	(0%)	0	(0%)	0	(0%)	0	(0%)	12	(100%)
NOS Subtype*	2	(22%)	1	(11%)	3	(33%)	1	(11%)	2	(22%)	0	(0%)	9	(100%)
**Total TETs**	**181**	**(64%)**	**31**	**(11%)**	**22**	**(0%)**	**5**	**(2%)**	**24**	**(8%)**	**20**	**(7%)**	**283**	**(100%)**

Supplementary Table 3 *- TNM stages and histologic WHO classifications of the TETs diagnosed in the cohort. The percentages represent the proportion of the histologic TET type diagnosed in TNM stage.*
**Not Otherwise Specified subtype.*

Histology and Resection Status

WHO Classification	Non-resected		R0		R1		R2		Total	
Neuroendocrine Tumour	0	(0%)	3	(75%)	1	(25%)	0	(0%)	4	(100%)
Thymic Carcinoma	17	(44%)	14	(36%)	5	(13%)	3	(8%)	39	(100%)
Thymoma	20	(8%)	173	(72%)	29	(12%)	18	(8%)	240	(100%)
A	2	(9%)	19	(86%)	1	(5%)	0	(0%)	22	(100%)
AB	3	(4%)	65	(86%)	5	(7%)	3	(4%)	76	(100%)
B1	2	(5%)	33	(75%)	5	(11%)	4	(9%)	44	(100%)
B2	4	(6%)	40	(65%)	13	(21%)	5	(8%)	62	(100%)
B3	1	(8%)	5	(36%)	2	(14%)	6	(43%)	14	(100%)
Metaplastic thymoma	0	(0%)	1	(100%)	0	(0%)	0	(0%)	1	(100%)
Micronodular Thymoma	0	(0%)	10	(83%)	2	(17%)	0	(0%)	12	(100%)
NOS Subtype*	8	(89%)	0	(0%)	1	(11%)	0	(0%)	9	(100%)
**Total**	**37**	**(13%)**	**190**	**(67%)**	**35**	**(12%)**	**21**	**(7%)**	**283**	**(100%)**

Supplementary Table 4 *– Resection status and histology of all TETs diagnosed in the cohort.*
**This category includes 8 patients, who did not undergo surgery for various reasons, and a single patient diagnosed with the extremely rare "sclerosing thymoma" (a type no longer present in the current WHO classification of thymomas), which was characterized by replacement of most of tumour tissue by extensive sclerosis and inconspicuous areas of preserved thymoma tissue that was unclassifiable.*

Autoimmune Comorbidities	Cases
**Nervous System**	**59 (21%)**
Maysthenia Gravis	53 (19%)
Alzheimer's Disease	1 (0.4%)
Autoimmune Encephalitis	1 (0.4%)
Isaac's Syndrome	1 (0.4%)
Multiple Schlerosis	1 (0.4%)
Parkinson's Disease	1 (0.4%)
Polyneuropathy	1 (0.4%)
**Endocrine System**	**12 (4%)**
Diabetes Type 1	2 (0.7%)
Grave's Disease	2 (0.7%)
Thyrotoxicosis	2 (0.7%)
Autoimmune thyroid disease	1 (0.4%)
Cushing's Syndrome	1 (0.4%)
Hashimoto's Disease	1 (0.4%)
Hyperparathyroidism	1 (0.4%)
Hypothyreosis	1 (0.4%)
Hypothyreosis, Adrenal Insufficiency	1 (0.4%)
**Immune System**	**11 (4%)**
Rheumatoid Arthritis	2 (0.7%)
Fibromyalgia	2 (0.7%)
Antiphospholipid Syndrome	1 (0.4%)
Morbus Bechterew	1 (0.4%)
Polymyalgia Rheumatica	1 (0.4%)
Psoriasis Arthropathy	1 (0.4%)
Raynaud's Syndrome	1 (0.4%)
Schleroderma	1 (0.4%)
SLE	1 (0.4%)
**Hematological System**	**8 (3%)**
Red Cell Aplasia	2 (0.7%)
Anemia	2 (0.7%)
Hemostatic Defect	1 (0.4%)
Spherocytosis	1 (0.4%)
Trombocytopenia	1 (0.4%)
Trombocytopenia, Anemia	1 (0.4%)
**Integumentary System**	**5 (2%)**
Dermatomyositis	1 (0.4%)
Lichen Aureus	1 (0.4%)
Psoriasis	1 (0.4%)
Unspecific Autoimmune Dermatologic Disease	1 (0.4%)
**Gastro-Intestinal System**	**4 (1%)**
Celiac's Disease	1 (0.4%)
Colitis Ulcerosa	1 (0.4%)
Collagen Colitis	1 (0.4%)
Entero Colitis	1 (0.4%)

Supplementary Table 5 *–Disorders with a suspected autoimmune pathogenesis in the cohort diagnosed before the date of TET diagnosis.*
